# Computational Lower Limb Simulator Boundary Conditions to Reproduce Measured TKA Loading in a Cohort of Telemetric Implant Patients

**DOI:** 10.3390/bioengineering11050503

**Published:** 2024-05-17

**Authors:** Chase Maag, Clare K. Fitzpatrick, Paul J. Rullkoetter

**Affiliations:** 1 DePuy Synthes, Warsaw, IN 46580, USA; cmaag@its.jnj.com; 2 Department of Mechanical and Biomedical Engineering, Boise State University, Boise, ID 83725, USA; clarefitzpatrick@boisestate.edu; 3 Center for Orthopaedic Biomechanics, Department of Mechanical and Materials Engineering, University of Denver, Denver, CO 80208, USA

**Keywords:** computational modelling, statistical modelling, total knee replacement

## Abstract

Recent advancements in computational modeling offer opportunities to refine total knee arthroplasty (TKA) design and treatment strategies. This study developed patient-specific simulator external boundary conditions (EBCs) using a PID-controlled lower limb finite element (FE) model. Calibration of the external actuation required to achieve measured patient-specific joint loading and motion was completed for nine patients with telemetric implants during gait, stair descent, and deep knee bend. The study also compared two EBC scenarios: activity-specific hip AP motion and pelvic rotation (that was averaged across all patients for an activity) and patient-specific hip AP motion and pelvic rotation. Including patient-specific data significantly improved reproduction of joint-level loading, reducing root mean squared error between the target and achieved loading by 28.7% and highlighting the importance of detailed patient data in replicating joint kinematics and kinetics. The principal component analysis (PCA) of the EBCs for the patient dataset showed that one component represented 77.8% of the overall variation, while the first three components represented 97.8%. Given the significant loading variability within the patient cohort, this group of patient-specific models can be run individually to provide insight into expected TKA mechanics variability, and the PCA can be utilized to further create reasonable EBCs that expand the variability evaluated.

## 1. Introduction

Total knee replacement (TKR) surgery is a common procedure that is used to treat osteoarthritis of the knee. The goal of TKR is to relieve pain, improve function, and restore range of motion. TKR is a very successful procedure, with most patients reporting significant improvement in their quality of life [1,2]. However, there is a small percentage of patients who experience complications following TKR [3,4]. Complications such as implant loosening, instability, and wear are often investigated through physical simulator testing. Several experimental knee simulators have been developed to understand the mechanics of implant design or alignment, for example, on joint mechanics, including kinematics/stability or wear. The Stanmore knee simulator is a fixtured testing rig (no cadaveric tissue is present) that is typically used to assess wear in the tibiofemoral (TF) joint [5]. More recently, the AMTI VIVO simulator (AMTI, Boston, MA, USA) was developed primarily as a fixtured test bed for wear evaluation, but the six-degree-of-freedom control has enabled many other studies of joint mechanics [6]. The VIVO has also been modified to include quadriceps loading for whole-knee joint testing [7]. Another class of physical simulators are Oxford-style rigs that frequently use cadaveric tissue and apply loading at a simulated hip and ankle, such as the Kansas knee simulator (KKS), an electrohydraulic whole-joint knee simulator [8].

However, physical experiments have associated time, labor, and financial expenses, which restrict the number of evaluations that can be feasibly performed. To address this limitation, computational simulators, namely, explicit dynamic finite element models, have been developed to complement these physical experiments. Computational models of the Stanmore knee simulator have been used to predict kinematics, wear, and contact mechanics [9,10]. Advancing beyond the Stanmore knee simulator, a computational model of the AMTI vivo joint simulator was developed and validated [11]. Halloran et al. developed a computational representation of the KKS, while Baldwin et al. adapted it to perform deep knee bend (DKB) and validated both patellofemoral (PF) and tibiofemoral (TF) kinematics [8,12,13]. Fitzpatrick et al. (2014) developed a proportional-integral-derivative (PID)-controlled lower limb model that improved upon prior computational KKS models by incorporating additional actuators to better reproduce the physiological loading or kinematics at the knee [14]. Computational constructs like these can be used to evaluate TKR performance in a variety of conditions, including those that are difficult or impossible to simulate experimentally. For example, computational models can be used to incorporate variability more efficiently in implant geometry or alignment, or perform population-based studies, as complementary physical experiments may require new fixturing, manufacturing of physical parts, or may be unable to accommodate the desired changes, such as with modification to ligament properties. Unfortunately, it is still relatively common to include only a single loading condition per activity, or a single implant alignment, essentially representing a single average patient. To address these limitations, researchers have recently been developing population-based analyses, including the impact of variation in loading conditions on implant wear simulation [15], or incorporating intersubject anatomical variation in assessing implanted patellofemoral joint kinematics and contact mechanics [16].

In our prior work, PID control was interfaced with dynamic finite element (FE) lower limb simulations to operate multiple actuators simultaneously and produce telemetrically measured in vivo loading conditions at the joint [14]. The PID-controlled model was able to successfully recreate the flexion angle, compressive joint load, medial–lateral (ML) load distribution or varus–valgus (VV) torque, internal–external (IE) torque, and anterior–posterior (AP) force for deep knee bend, chair rise, stance-phase gait, and step-down activities. The external actuator loading conditions developed for this patient can be subsequently used in further studies of implant mechanics, but again only represent a single patient.

The objective of the current study is to further develop external boundary conditions for a cohort of nine telemetric TKA patients [17] using the prior PID-controlled lower limb model [14], and to quantify the variability in external boundary conditions required for simulation.

## 2. Methods

### 2.1. Lower Limb Finite Element Model

**Figure 1:**
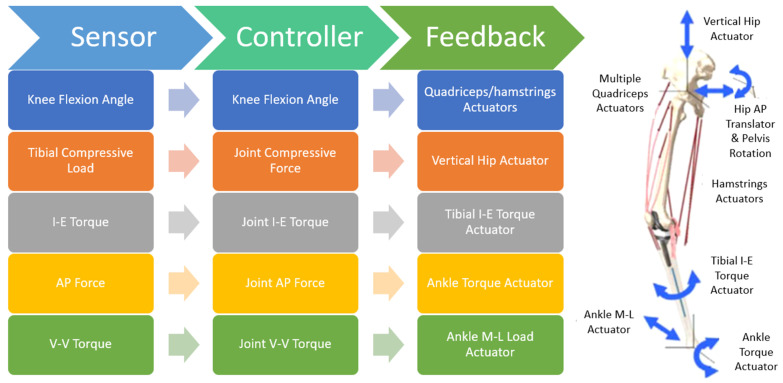
Schematic of the control algorithm and actuation of the lower limb simulator, with hamstring muscles and physiological quadricep muscle paths. Blue arrows denote axis of applied actuator loading.

A previously developed FE model of the lower limb with integrated PID control was used in this study, and for completeness will be briefly described here [14]. The lower limb model includes femoral, tibial, patellar, and pelvic bones, TKR implants (INNEX^TM^ System, Type FIXUC; Zimmer GmbH, Winterthur, Switzerland), two-dimensional TF ligaments, the patellar ligament, and quadricep and hamstring muscles (Figure 1). TF soft tissue included representations of the medial and lateral collateral ligaments, the popliteofibular ligament, the anterior lateral capsule, and the posterior capsule [12,16,18]. These structures, and the patellar ligament, were modeled with two-dimensional fiber-reinforced membrane elements, with ligament pre-tension, stiffness, and attachments sites optimized to match previously published cadaveric laxity data [16]. The quadricep muscles were represented by four muscle bundles (rectus femoris, vastus intermedius, vastus medialis, and vastus lateralis) with lines of action of each muscle bundle estimated from the Visible Human dataset [19]. The hamstring muscles were represented by four point-to-point muscle actuators (semimembranosus, semitendinosus, and long and short heads of the biceps femoris).

The ankle was free in all rotational DOFs and ML translation and fixed in AP and superior–inferior (SI) translational DOFs. The hip was free in all rotational DOFs and SI translation, fixed in ML translation, and AP hip motion was kinematically prescribed. Actuators in the model were used to apply a vertical hip load to the femoral head, muscle loads to the quadriceps and hamstrings, and ML load, IE, and flexion–extension torque to the ankle. TF and PF joints were kinematically unconstrained in all 6-DOFs. Knee flexion in the lower limb model was achieved through a combination of vertical hip force and muscle force. Vertical hip force and hamstring force served to flex the knee, while the quadriceps force extended the knee. Knee flexion was controlled by a balance of these two factors. Using a PID controller to calculate the required actuator loads, vertical hip force was applied to achieve a target TF compressive force profile, while quadricep (knee flexion) and hamstring (knee extension) forces were applied to achieve a target knee flexion profile [14]. Additionally, controlled actuator loads created specified IE, AP, and VV TF joint loads.

### 2.2. Telemetric Implant Patient Data

**Figure 2:**
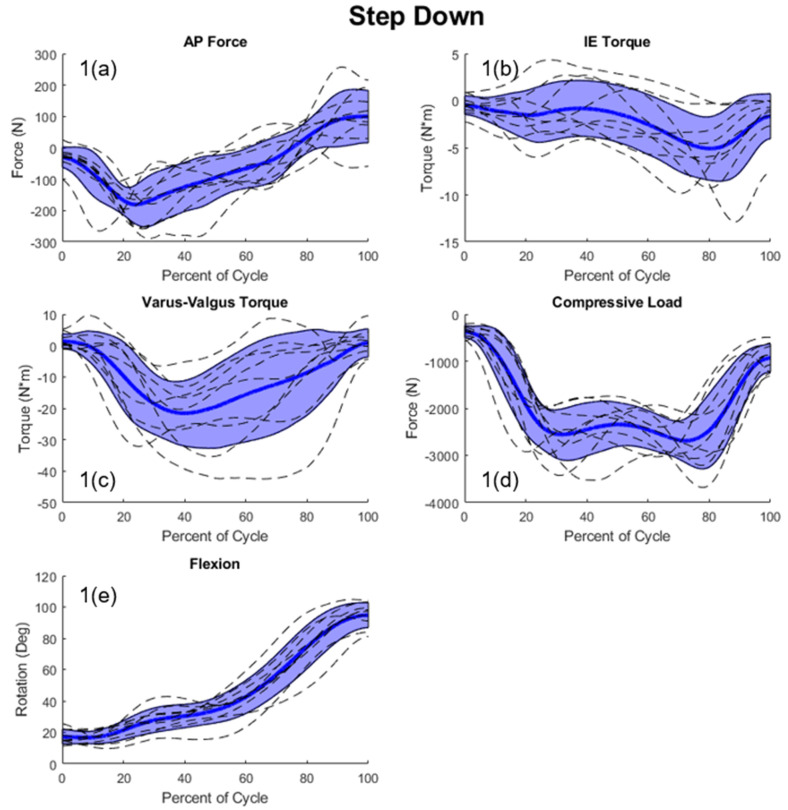

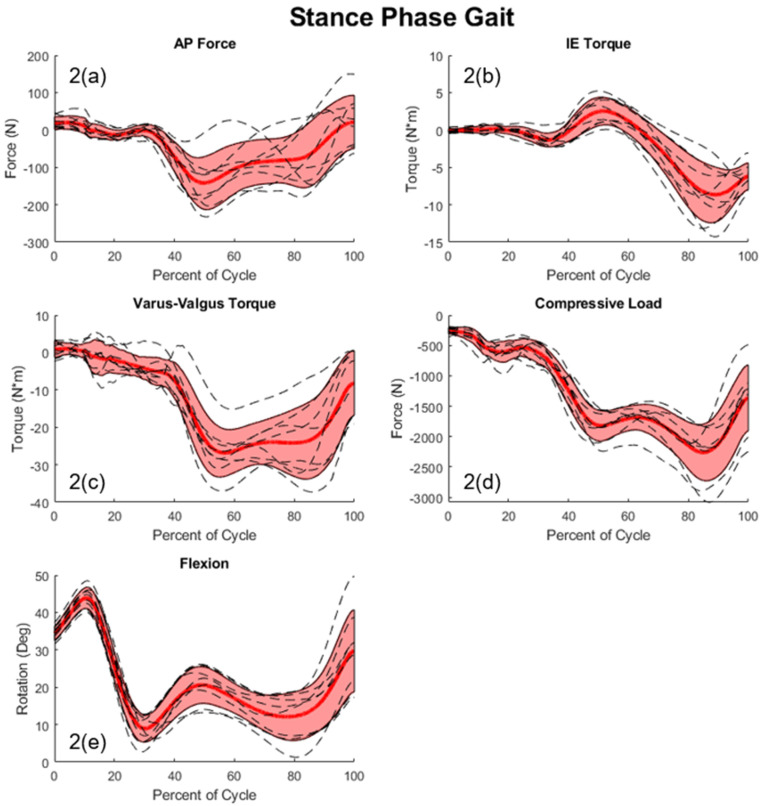

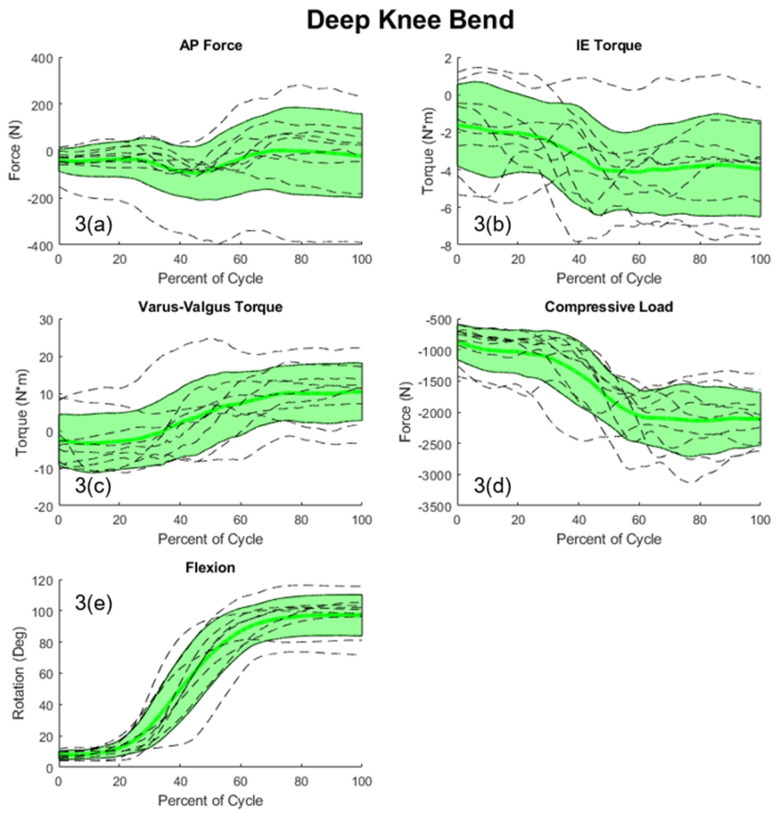
Joint loads for all patients (dashed lines) with shaded regions representing +/−1 standard deviation of the mean; (**1(a)**–**1(e)**) step-down kinematics = blue; (**2(a)**–**2(e)**) stance phase gait kinematics = red; (**3(a)**–**3(e)**) deep knee bend kinematics = green; (forces with respect to the tibia: +anterior; +internal; +varus; and −compressive).

Telemetric implant and kinematic data for nine TKA patients were adopted from the Orthoload database [15]. This provided 6-DOF loads acting on the tibial tray, as well as knee flexion, pelvic rotation, patient weight and height, and implant orientation. Three activities were included: DKB, step-down (SD), and stance-phase gait (Figure 2). Data was available for all nine patients in DKB and SD, however one patient was missing gait data. Raw data for the hip AP motion was not included in the original dataset; however, this data was extracted from a video of the patients using manual post-processing. This was accomplished by finding the hip center and extracting its relative position to the knee center and ankle center for each frame of the video. Patient profiles for each activity were created containing flexion, compressive and AP load, VV and IE torque, pelvic rotation, and hip AP motion.

### 2.3. Optimization of Control Parameters and Estimation of Actuator Loading

As described previously, the PID control parameters for each patient were tuned independently. The proportional and integral gains for each axis were determined by uniformly varying these values, choosing the best fit as the lowest root mean square error (RMSE) between the model result and desired implant loading [14].

As the hip motion measured for the patient cohort included significant variability, two versions of each patient’s data were analyzed to evaluate the impact of this variation: one set with patient- and activity-specific pelvic rotation and hip AP motion and another set with only activity-specific pelvic rotation and hip AP motion. Activity-specific-only data pertain to average motion across all patients. Applying each set independently and tuning the PID to best fit the desired tibial loads enabled an understanding of the impact of patient-specific hip movement on the ability to properly reproduce patient loading. Once each model was tuned to best represent patient loading, the RMSE between the target and achieved loading was calculated for each axis. The average and standard deviation RMSE across all patients were calculated for both data sets to compare the control schemes.

### 2.4. Principal Component Analysis

To understand interdependency between the actuator loads, principal component analysis (PCA) was applied to identify any relationships between actuator loads. The input data for the PCA consisted of the actuator load profiles for each patient, across the activity cycle. The primary modes of variation (PC scores) were extracted and visualized to explain how loading profiles varied with one another. This data allowed us to generate new loading profile instances for all DOFs that maintained relationships between actuator loads, with the potential to sample for future studies based on this population.

## 3. Results

**Figure 3:**
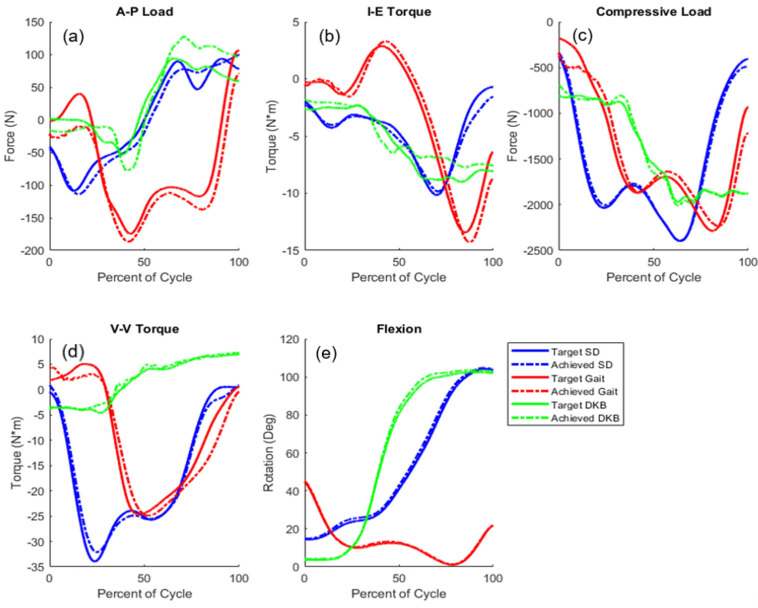
Representative subject target and achieved joint loads during SD (Patient 7), gait (Patient 3), and DKB (Patient 8)—subject selected based on RMSE most closely matching average; (**a**) anterior–posterior (A–P) force; (**b**) internal–external (I–E) torque; (**c**) compressive load; (**d**) varus–valgus (V–V) torque; (**e**) flexion; (forces with respect to the tibia: +anterior; +internal; +varus; −compressive; target joint load as measured by the telemetric implant; achieved applied joint load in the FE model, as generated via the PID-controlled actuators at the hip, ankle, and muscles).

Calibration of external simulator actuation to achieve the measured patient-specific joint loading and motion was completed for all nine patients. The RMSE differences between joint load targets and those achieved by the controlled simulations were calculated for all patients and activities (Table 1; Figure 3). Including patient-specific hip AP motion and pelvic rotation reduced the RMSE by an average of 28.7% compared with using activity-specific pelvic rotation and hip AP motion. The largest reduction was seen in the DKB activity, where the RMSE went from 39.95 N to 31.17 N, 1.15 N-m to 0.65 N-m, 189.07 N to 44.37 N, 1.67 N-m to 0.81 N-m, and 1.69° to 1.63°, for AP load, IE torque, compressive load, VV torque, and flexion angle, respectively (Table 1). Patient-specific hip and pelvic actuation was most impactful in reducing RMSE in compressive load in DKB (reduced by 76%). Overall, RMSE errors for the patient-specific hip and pelvic motion models were 28.56 N, 0.65 N-m, 97.72 N, 1.88 N-m, and 1.44° for AP load, IE torque, compressive load, VV torque, and flexion angle, respectively.

**Figure 4:**
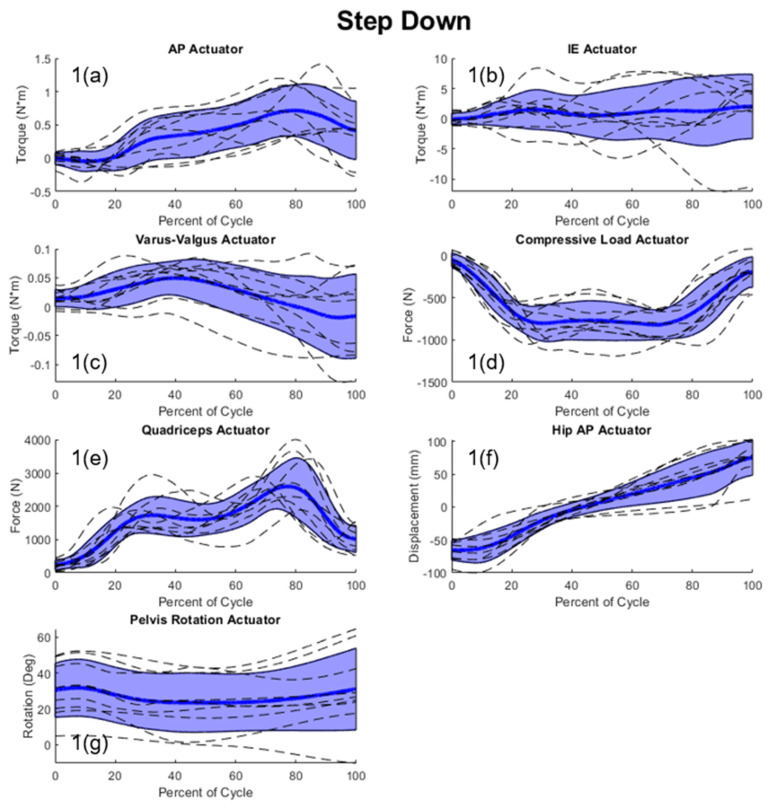

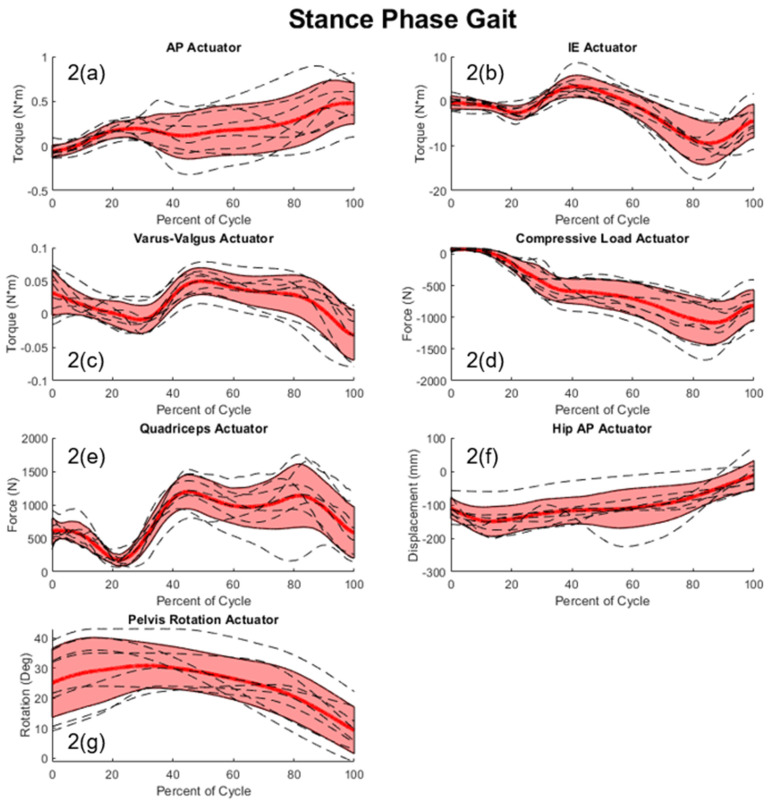

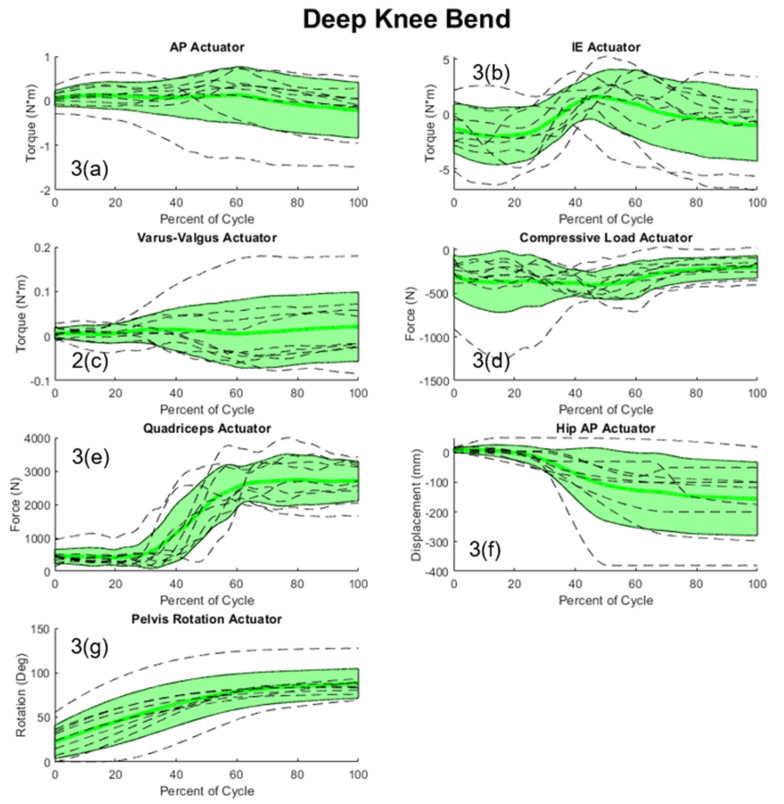
Joint simulator actuator input for all patients (dashed lines) with shaded regions representing +/−1 standard deviation of the mean; (**1(a)**–**1(g)**) step-down actuator inputs = blue; (**2(a)**–**2(g)**) stance phase gait actuator inputs = red; (**3(a)**–**3(g)**) deep knee bend actuators inputs = green; (forces with respect to the tibia: +anterior (ankle extending); +internal; +varus; and −compressive).

Significant variability in the measured joint-level loading data was seen for this patient cohort, and this resulted in a correspondingly large variability in the simulation actuators. The average standard deviation of the measured joint loading across patients was 121 N, 3.52 N-m, 12.3 N-m, 655 N, and 15.4° for AP, IE, VV, compressive load, and flexion, respectively. Peak standard deviations were seen in the measured joint load of the SD activity (809 N) and the AP load of the DKB activity (185 N). The actuator response to this variability in the joint-level loading created an average standard deviation of 467 N, 4.69 N-m, 330 N, 0.064 N-m, 772 N, 73.2 mm, 20.3° for the AP, IE, compressive load, VV, quadriceps load, hip AP, and pelvic rotation actuators, respectively (Figure 4). Peak actuator standard deviations were seen in the quadriceps actuator (953 N) in SD, and in the AP loading actuator (651 N) during the DKB activity (Figure 4).

**Figure 5:**
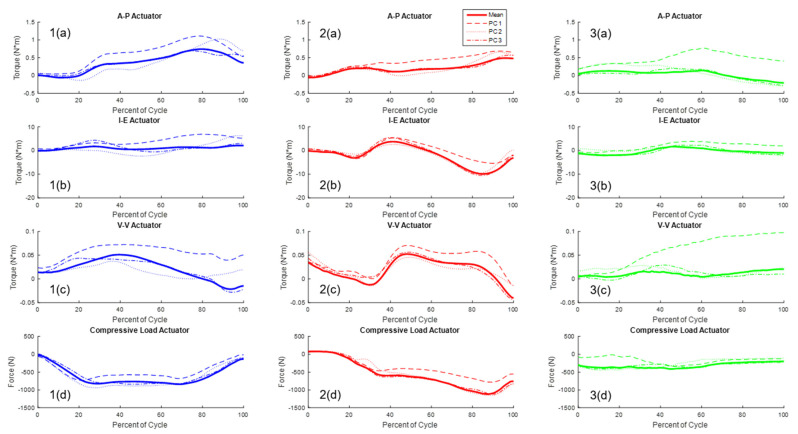
Effect of primary PC modes on the mean actuator load; (**1(a)**–**1(d)**) step-down PC modes = blue; (**2(a)**–**2(d)**) stance phase gait PC modes = red; (**3(a)**–**3(d)**) deep knee bend PC modes = green; (forces with respect to the tibia: +anterior (ankle extending); +internal; +varus, and −compression).

Principal component analysis (PCA) was employed to understand interdependencies between simulator actuation (Figure 4) using the more accurate actuator loading derived from including the patient-specific hip AP and pelvic motion. The PCA showed that the first three PCs accounted for approximately 97.8% of the variability in the actuator loading. PC1 (responsible for 77.8% variation) indicated a relationship between vertical hip load, and the AP and IE force required to achieve target joint loads, with increasing compressive load at the hip actuator, increased ankle flexion–extension torque and tibial IE torques are required to achieve target joint loads (Figure 5). PC2 and PC3 accounted for 15.8% and 4.2%, respectively, of the variability in the actuator loading.

## 4. Discussion

This work developed external simulator loading conditions required to reproduce joint-level loading for a cohort of nine patients with telemetric implants. This was completed through the use of a previously developed PID-controlled lower limb FE model [14] coupled with the Orthoload database [15]. Controllers were integrated with FE model actuators and used to determine the simulator actuator loading required to best reproduce the measured joint-level loading. The resulting patient-specific models with calibrated loading conditions are a forward-dynamic platform for use in subsequent knee implant design and evaluation, including associated mechanics of interest, such as the impact of post-operative ligament balancing. Given the ease with which computational boundary conditions are imposed, these nine patient-specific models can be run directly, or a larger probabilistic computational study can be performed through sampling within the PCA representation to evaluate a larger range of potential boundary conditions.

Although based on a small cohort of patients, the joint-level loading varied substantially within the experimental dataset, with peak standard deviations up to 809 N. Although a wear test may be reasonably performed based on an overall ‘worst-case’ loading condition for a composite of activities, evaluations of joint mechanics are better served through an understanding of the potential response in the population. This work represents an evolution in that direction, as well as a process for continuing to add patient data as it becomes available.

Tuning of the controllers enabled a good reproduction of the desired loading conditions, especially with the inclusion of patient-specific AP position and pelvic rotation. The patient-specific data substantially improved joint loading accuracy compared with using generic, activity-specific values for these variables. The resulting average RMSE for the patient-specific cohort was comparable with that of the previously published RMSE for a single patient (17 N, 0.8 N-m, 65 N, and 0.5°, for AP load, I–E torque, compressive load, and flexion, respectively) [14] and was significantly improved over other previously published boundary condition studies [20,21]. Given the sensitivity of the joint-level loading to the hip AP and pelvic rotation inputs, physical lower limb simulators may consider including these as additional actuators or at least variable static alignments in the future.

Peak actuator standard deviations were found in the quadriceps (approaching 1000 N) and AP (651 N) actuators, which were, surprisingly, larger than the vertical loading actuator variability. Further study should ideally incorporate patient whole-body kinematics and habitus for this cohort to better understand how individual mechanics drive these variations.

The PCA identified preliminary interdependencies between simulator boundaries conditions (actuator loads) across activities. Notably, the increased compressive load at the vertical hip actuator was associated with larger ankle flexion torque and tibial internal torque. This analysis can provide guidance for new combinations of simulator boundary conditions that are compatible with one another to create physiological joint loading conditions. While we have established this methodology here, a more comprehensive dataset will be necessary to verify if these relationships hold across a broader sample of the patient population.

There are several study limitations that should be considered. The sample size in this study was relatively small (n = 9), which does limit the ability to generalize to the full population. Patient-specific bony geometry was not available, and hence generic attachment sites, measured in prior cadaveric work [19], were applied across all simulations. Similarly, while the dataset did provide some patient demographics (body weight and height) and joint alignment, it did not have information on the pre-/post-implantation soft tissue state. As such, the soft tissue representation was adopted from prior work [19] across all simulations, although variable soft tissue conditions could be included in a future probabilistic study. The study focused on only three activities of daily living (gait, SD, and DKB). However, these activities included both the most common (gait) and more demanding (SD, DKB) tasks, and so provided a reasonable activity spectrum across which to evaluate TKR mechanics. Although the implanted position was known, the post-operative ligamentous state was not, and the ligament representation was consistent for each patient. Additionally, this study did not have access to any PF data for the patients nor a line of action of the quadriceps during activity, and representation of the quadricep and hamstring muscles was simplified to be controlled as a group rather than individual muscles.

This study developed patient-specific external boundary conditions for TKA simulations. The approach utilized a PID-controlled lower limb FE model coupled with the Orthoload database to replicate measured joint loads in nine patients. The resulting models can be used to evaluate implant designs and associated mechanics, including, but not limited to, alignment techniques or post-operative ligament balancing. Patient-specific data significantly improved model accuracy, as shown by including the hip AP position and pelvic rotation. These models achieved a similar RMSE to previously reported single-patient studies, while improving upon them by increasing patient population and associated loading variability. Analysis of actuator loads revealed significant variations across patients, with the highest in quadriceps and AP actuators. PCA identified interdependencies between boundary conditions, enabling broader probabilistic studies beyond the variability included in this patient cohort.

## Figures and Tables

**Table 1:** Average and standard deviation of RMSE difference between target and achieved joint loads with and without patient-specific hip AP motion.

	Average Hip AP and Pelvic Rotation					Activity and Patient-Specific Pelvic Rotation and Hip AP Motion				
	AP Force	IE Torque	Compressive Load	VV Torque	Flexion	AP Force	IE Torque	Compressive Load	VV Torque	Flexion
Gait	52.22 ± 45.16 N	0.89 ± 0.90 N×m	223.81 ± 118.80 N	4.20 ± 3.95 N×m	0.69 ± 0.16 Deg	29.35 ± 11.81 N	0.74 ± 0.20 N×m	151.54 ± 55.48 N	2.77 ± 0.64 N×m	0.61 ± 0.09 Deg
SD	23.46 ± 22.71 N	0.81 ± 0.47 N×m	185.48 ± 46.73 N	2.69 ± 0.61 N×m	2.11 ± 0.39 Deg	25.16 ± 18.69 N	0.57 ± 0.34 N×m	97.26 ± 53.82 N	2.07 ± 0.58 N×m	2.08 ± 0.45 Deg
DKB	39.95 ± 29.40 N	1.15 ± 1.22 N×m	189.07 ± 98.88 N	1.67 ± 0.73 N×m	1.69 ± 0.40 Deg	31.17 ± 19.63 N	0.65 ± 0.54 N×m	44.37 ± 22.03 N	0.81 ± 0.32 N×m	1.63 ± 0.37 Deg

## Data Availability

The datasets presented in this article are not readily available because of commercial restrictions.
